# Enhanced Mechanical Performance of Resin-Infused 3D-Printed Polymer Lattices

**DOI:** 10.3390/polym17081028

**Published:** 2025-04-10

**Authors:** Jakub J. Słowiński, Maciej Roszak, Mikołaj Kazimierczak, Grzegorz Skrzypczak, Maksymilian Stępczak

**Affiliations:** 1 Department of Mechanics, Materials and Biomedical Engineering, Faculty of Mechanical Engineering, Wroclaw University of Science and Technology, Smoluchowskiego 25, 50-372 Wroclaw, Poland; jakub.slowinski@pwr.edu.pl (J.J.S.); maciej.roszak@pwr.edu.pl (M.R.); mikolaj.kazimierczak@pwr.edu.pl (M.K.); 2 Astriva Sp. z o.o., Przybysławice 5A, 63-440 Raszków, Poland; greg.skrzypczak@gmail.com

**Keywords:** additive manufacturing, composites, compressive strength, mechanical properties, numerical analysis

## Abstract

Fused deposition modelling (FDM) technology provides a flexible and cost-effective solution for the manufacture of polymer components, enabling the precise design of structures and the incorporation of a variety of composite materials. Its development is confirmed by numerous studies on fibre reinforcements (e.g., GFRP and CF) and thermosetting resin modifications, resulting in improved impact strength and fracture toughness and increased thermal stability of products. The final mechanical properties are significantly influenced by processing parameters (e.g., fill density, layer height, and printing speed) and internal geometry (e.g., lattice structures), which can be further optimised by numerical analyses using constitutive models such as the Johnson–Cook model. The focus of the study presented here is on the fabrication of composites from FDM dies filled with F8 polyurethane resin. Filaments, including PETG carbon and PETG, were tested for potential applications with the resin. A static compression test, supported by numerical analysis using the Johnson–Cook model, was carried out to identify key mechanical characteristics and to predict the material’s behaviour under different loading conditions. The results indicate that these structures exhibit numerous potential delamination planes and voids between filament paths, leading to relatively low maximum stress values (*σ*_*m*_ ≈ 2.5–3 MPa). However, the impregnation with polyurethane resin significantly enhances these properties by bonding the layers and filling the pores, resulting in a more homogeneous and stronger composite. Additionally, numerical simulations effectively captured key aspects of structural behaviour, identifying critical stress concentration areas, particularly along the side walls and in regions forming triangular stress zones. These findings provide valuable insights into the potential of resin-filled FDM structures in engineering applications, demonstrating their improved performance over purely printed samples.

## 1. Introduction

Fused deposition modelling (FDM) is a versatile and cost-effective additive manufacturing technology that has revolutionized the production of polymer-based components. Its ability to fabricate complex geometries, combined with its ability to incorporate diverse composite materials, positions it as a crucial method for modern engineering and industrial applications. This technology enables the customization of mechanical and functional properties through the careful optimization of materials, parameters, and post-processing techniques, making it an ideal platform for developing innovative materials.

The use of FDM for creating composites has gained significant attention, as it offers unique opportunities for combining thermoplastics with reinforcing agents or functional fillers. Studies have demonstrated enhanced fracture toughness in epoxy composites by utilizing 3D-printed PLA Bouligand structures, exemplifying the role of FDM in achieving superior material performance [1]. Hybrid composites such as PLA/GFRP (polylactic acid/glass fiber reinforced polymer), produced through FDM and filament winding, have shown exceptional crashworthiness and mechanical robustness, highlighting the synergistic potential of hybrid fabrication techniques [2]. Similarly, FDM-manufactured sandwich composite plates with customized core geometries have exhibited improved low-velocity impact resistance, underscoring the importance of geometry optimization [3]. Material and process innovations in FDM, such as incorporating biocomposites and environmentally friendly fillers, have further expanded its applications. For example, studies on biomass-filled PLA demonstrate both high printability and mechanical performance, supporting the adoption of sustainable materials [4]. Fiber-reinforced composites, including glass and carbon fibers, have been found to significantly enhance tensile and compressive properties when used in FDM applications [5,6,7]. Epoxy-infused composites, in particular, have demonstrated superior stiffness and thermal stability compared to standard thermoplastics, illustrating the potential of resin-filled structures [8,9].

Processing parameters such as infill density, layer height, and printing speed play a pivotal role in determining the mechanical performance of FDM-printed parts. Numerous reviews emphasize the correlation between these parameters and the structural integrity of composites [10,11,12]. Beyond parameters, the integration of thermoset resins like epoxy into FDM-manufactured thermoplastic matrices further enhances the mechanical properties, offering a balance between rigidity and flexibility [13,14]. FDM also provides opportunities for advanced numerical analysis and simulation, with models like the Johnson–Cook model enabling accurate predictions of material deformation under stress. Such approaches have proven invaluable in optimizing material combinations and designs [15,16]. Further to this, the exploration of lattice geometries and core-shell structures has revealed new avenues for impact resistance and energy absorption in composites, addressing critical demands in aerospace and automotive industries [17,18]. As composite material research evolves, the integration of flexible and rigid elements within FDM designs has shown promise. For example, thermoplastic–polyurethane (TPU) composites and other elastomeric materials exhibit enhanced flexibility, making them suitable for specialized applications [19,20]. Coupled with thermoset resins, these materials provide unprecedented customization of mechanical properties [21,22].

This work examines the potential of resin-infused, open-cell lattice structures as a means to enhance mechanical performance while preserving the inherent mass-saving and design flexibility of 3D-printed matrices. By leveraging FDM-printed PETG (polyethylene terephthalate glycol) and PETG carbon filaments flooded with polyurethane resin F8, we explore a hybrid strategy that capitalizes on precisely engineered porosity, permitting tailored stiffness, localized reinforcement, and controlled energy absorption. Compressive strength tests, supported by Johnson–Cook-based numerical analyses, provide insights into the feasibility and benefits of partial or selective resin infiltration in critical load-bearing zones. Building on previous advances, this study aimed to evaluate the performance and application potential of such hybrid composites in aerospace, automotive, and biomedical contexts, with particular focus on achieving an effective balance between weight reduction, mechanical adaptability, and design freedom.

## 2. Materials and Methods

### 2.1. Characteristics of Printed Materials

PETG is a thermoplastic copolyester with mechanical properties between those of PLA and ABS (acrylonitrile butadiene styrene). It is characterized by high impact resistance, good interlayer adhesion, and relatively low shrinkage during printing. The chemical modification of PET by adding glycol reduces its brittleness and increases flexibility, making it more resistant to cracking and impacts compared to standard PET (polyethylene terephthalate). Additionally, this material has low moisture absorption and good resistance to chemical agents such as acids and bases, making it suitable for applications in high-humidity environments and exposure to external factors.

PETG carbon is a composite printing material, a modified version of polyethylene terephthalate copolyester with the addition of carbon fibers. It displays high stiffness and mechanical strength while maintaining good flexibility compared to pure technical polymers. The addition of carbon fibers increases the elastic modulus and improves creep resistance, making it an attractive choice for applications requiring high dimensional stability. The mechanical properties of PETG carbon provide higher resistance to deformation under static and dynamic loads compared to conventional PETG, as well as enhanced resistance to high temperatures. This material is chemically resistant, has low moisture absorption, and is suitable for applications in humid environments. Additionally, it exhibits good interlayer adhesion in the 3D printing process using the FDM method, resulting in high uniformity and structural integrity of finished components.

PU resin F8 (polyurethane resin) is a cold-curing material used in applications requiring increased flexibility and vibration damping. It is a resin with relatively low viscosity, allowing the effective filling of porous structures and its use in composites with complex geometries. Once cured, it forms a material with high wear resistance and good adhesion to various substrates, including plastics and metals. A key feature of F8 resin is its ability to deform elastically while maintaining structural integrity, enabling energy absorption during dynamic loads. It is characterized by good resistance to atmospheric conditions and chemical agents, making it a suitable choice for applications requiring high durability in harsh environmental conditions.

Thanks to its properties, PU F8 resin can significantly enhance the mechanical strength of 3D-printed composites. Its low viscosity allows it to effectively fill pores and microvoids within FDM structures, resulting in a more homogeneous and consolidated material. By filling the gaps between filament layers, the resin mitigates typical FDM weaknesses such as delamination and local buckling, improving cohesion and load-bearing capacity. Additionally, the elastic nature of the cured resin contributes to energy dissipation under dynamic loads, further increasing the material’s durability and resistance to failure.

The material properties of plastics used in this study are summarized in Table 1.

### 2.2. Preparation of Samples

Samples for quasi-static compression testing were prepared in accordance with the guidelines of ISO 844—Rigid cellular plastics—Determination of compression properties [27], which specifies the requirements regarding the geometry, dimensions, and test conditions for thermoplastic polymer materials in compression strength testing. To ensure reliable and repeatable results, each sample was manufactured using the 3D printing (FDM) method on an Original Prusa MK4 printer (Prusa Research, Prague, Czech Republic) maintaining uniform process parameters using PrusaSlicer 2.8.1 software (Prusa Research, Prague, Czech Republic). During the printing process, the nozzle and bed temperatures were set to 250 °C and 90 °C, respectively, for PETG and 225 °C and 90 °C for PETG carbon.

**Figure 1:**
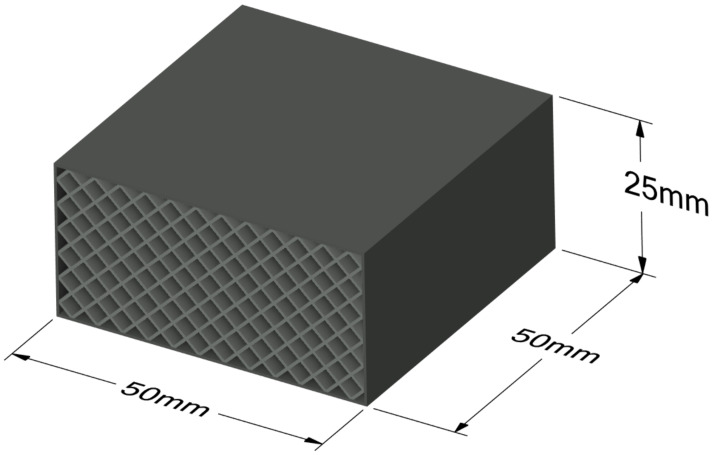
Dimensions of samples used in compressive strength tests and numerical simulations.

According to the recommendations outlined in ISO 844 (Clauses 7 and 8), the samples were shaped as rectangular blocks (Figure 1) with a square cross-section of 50 mm × 50 mm and a height of 25 mm, ensuring a height-to-width ratio of 2:1. This geometry, compliant with the standard, enables uniform stress distribution during compression and facilitates deformation measurements. The parallelism of the top and bottom surfaces was ensured by means of light sanding after printing, achieving a parallelism tolerance of ±0.05 mm (ISO 604:2002, Clause 5.4) [28].

**Figure 2:**
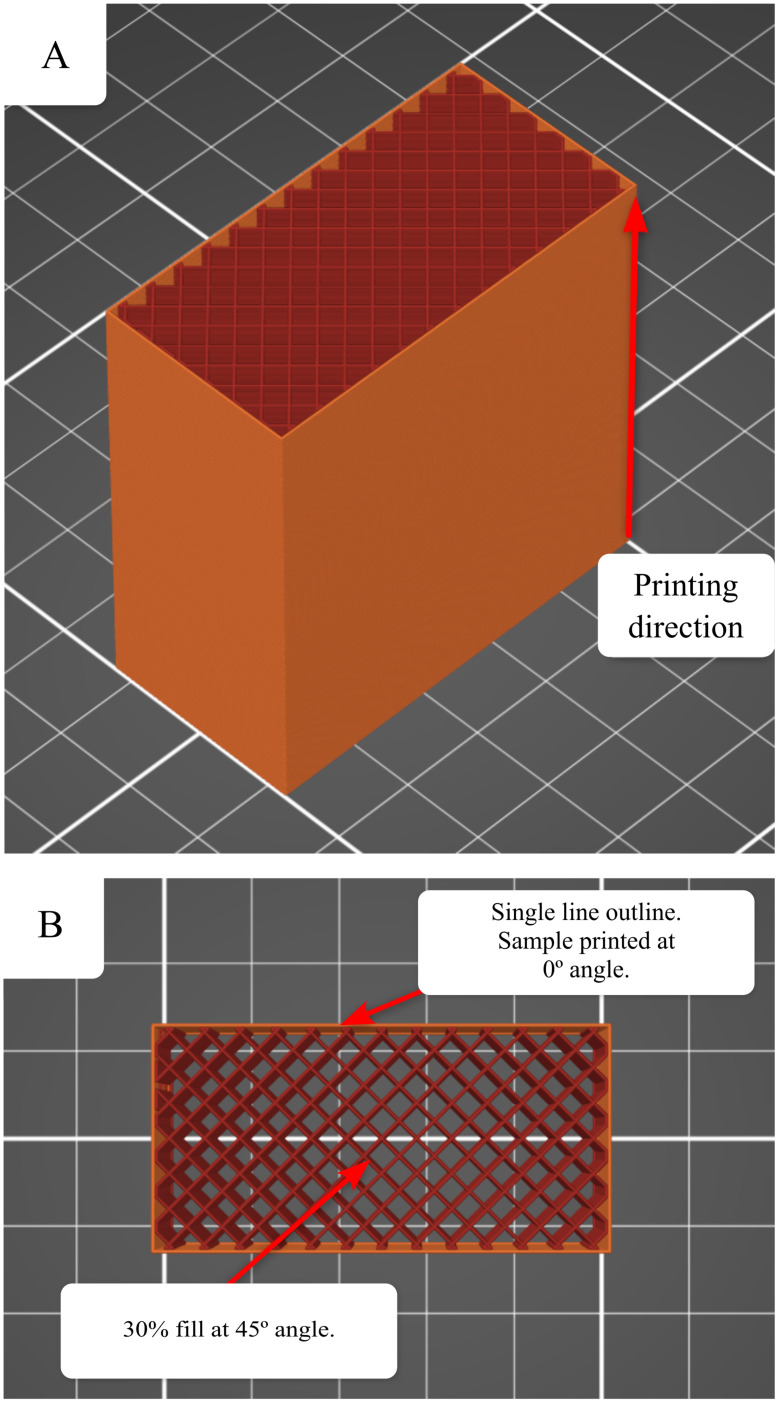
Sample file in the PrusaSlicer 2.8.1 software used for printing: (**A**) isometric view; (**B**) top view.

All samples were printed in a vertical orientation so that the layers were arranged perpendicular to the loading direction. This approach minimized the anisotropy of mechanical properties, which is characteristic of FDM-manufactured parts, and improved result repeatability. The printing process parameters were standardized: the layer height was 0.2 mm, and 30% infill ensured full sample density, eliminating internal voids. Each sample had a single perimeter to minimize the influence of perimeters on the behavior of the infill itself. The printing parameters are presented below (Figure 2).

The choice of a 30% infill was to simulate realistic structural conditions in which weight and material usage are optimized—common practice in engineering applications using FDM. This level of infill provides a balance between mechanical performance and production efficiency. More importantly, a 30% infill creates a porous lattice with enough internal space to clearly observe and evaluate the impact of resin impregnation on mechanical enhancement.

The grid was set as chosen infill due to its characteristics as a lattice structure. One of the primary advantages of lattice structures lies in their ability to absorb and dissipate energy through controlled deformation mechanisms. When subjected to impact or compressive loads, these structures undergo localized buckling, bending, and the progressive collapse of individual cells. This behavior enables them to spread and reduce the intensity of stress concentrations, thereby protecting more critical components of a system. In addition to energy dissipation, lattice structures are inherently lightweight due to their open, porous architecture. This reduction in material volume leads to a high strength-to-weight and stiffness-to-weight ratio, which is especially beneficial in fields where mass reduction is a key performance factor.

**Figure 3:**
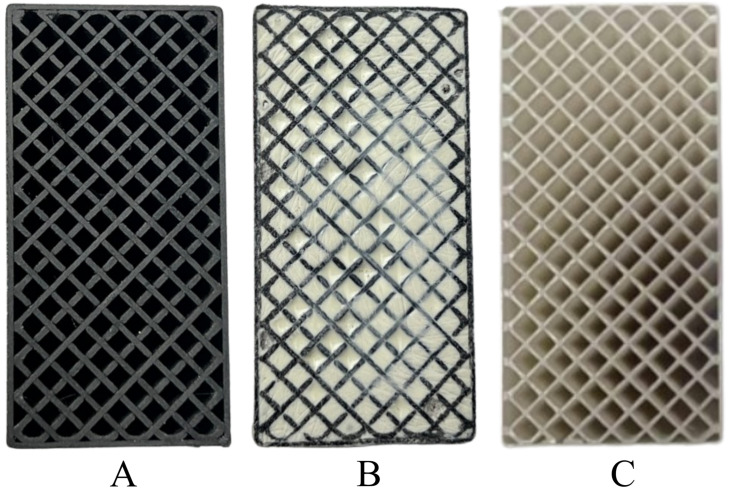
Top view of the prepared samples: (**A**) PETG carbon with 30% infill; (**B**) PETG carbon sample filled with resin; (**C**) PETG with 30% infill.

After printing 10 samples for each of the tested filaments, 5 of them were filled with polyurethane resin F8. The mass ratio of the resin-to-hardener mixture was 10:4. The prepared mixture was thoroughly stirred and then poured into the empty spaces within the structures printed using the FDM method. After pouring, the samples were placed on a vibrating table to eliminate voids caused by trapped air between the infill walls. Before testing, all samples were conditioned under controlled conditions of (23 ± 5 °C) and 50% ± 5% relative humidity for at least 24 h, in accordance with ISO 291:2008 [29] (and ISO 844, Clause 7.4), to ensure material property stabilization and result comparability. After conditioning, each sample was measured using calibrated measuring instruments to verify dimensional compliance with the standard requirements (i.e., a width of 50 ± 0.2 mm, a height of 25 ± 0.2 mm, and ensured parallelism of the top and bottom surfaces). The prepared samples, with their rectangular geometry and uniform manufacturing parameters, formed the basis for further quasi-static compression testing, enabling the acquisition of representative results regarding the elastic modulus, yield strength, and compressive strength of the tested materials (Figure 3).

**Figure 4:**
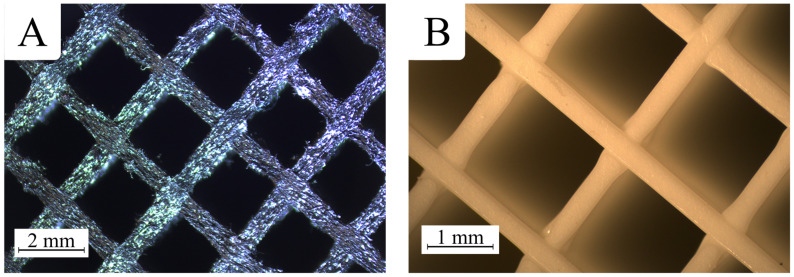
Comparison of printed structures: (**A**) PETG carbon; (**B**) PETG.

To assess the quality of the manufactured samples and verify their uniformity, they were examined under a stereoscopic microscope, OptaTech SK (OptaTech, Warsaw, Poland). A noticeable difference in the topography was observed between the raw filament and the finished prints (Figure 4).

**Figure 5:**
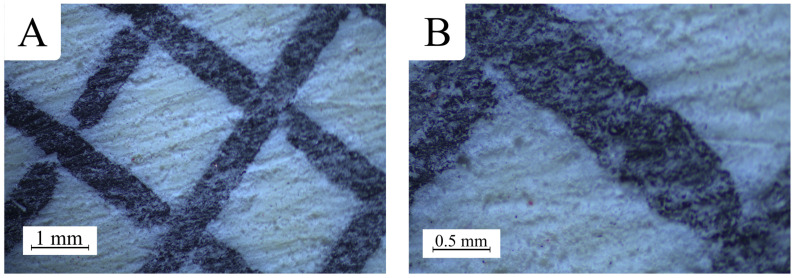
Microscopic view of the resin-to-print wall interface: (**A**) close-up; (**B**) close-up of the surface.

It can be assumed that the observed microscopic surface roughness of PETG carbon prints is mainly due to the introduction of carbon fibers into the PETG base matrix (Figure 4). These fine particles may cause local disturbances in the rheology of the molten polymer, which in turn promotes the formation of micro-irregularities and a significant increase in micro-scale roughness. It is also possible that the intensified wear of the printing nozzle—due to the abrasive properties of the fibers—prevents as smooth and uniform material extrusion as in the case of pure PETG. As a result, PETG carbon prints may exhibit characteristic "grainy" structures visible under high magnification, even though they often appear relatively uniform and matte on a macro scale (Figure 5).

Microscopic images show partial distortions in the areas where the print walls intersect. In these regions, excess material is visible, resulting from the printing settings.

### 2.3. Testing of Materials in Compressive Strength Test

**Figure 6:**
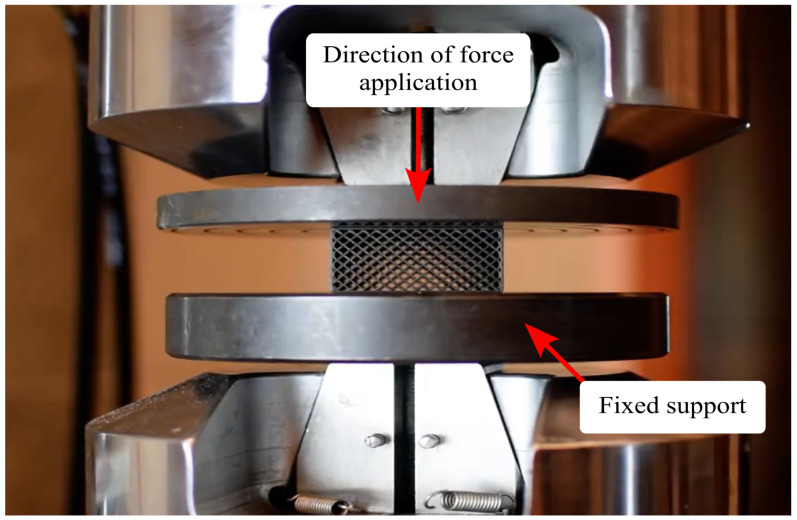
A PETG carbon sample with 30% infill mounted on the used MTS 810 testing machine.

Mechanical property testing for compressive strength was conducted in accordance with ISO 844, which defines the methodology for evaluating the compressive parameters of rigid cellular plastics. The procedure included sample preparation, application of axial loading under controlled conditions, and analysis of the obtained mechanical characteristics. The test samples were rectangular prisms with a square base and dimensions of 50 × 50 × 25 mm, meeting the normative requirements for geometry and proportions used in compression tests. The contact surfaces were precisely prepared to minimize the influence of irregularities on measurement results. The experiments were carried out using an MTS 810 (MTS Systems Corporation, Eden Prairie, MN, USA) universal testing machine equipped with rigid, parallel compression plates, with one plate remaining stationary while the other moved at a controlled displacement rate (Figure 6).

During the tests, the samples were subjected to quasi-static axial loading with a deformation rate of 10% of the initial sample thickness per minute (i.e., 2.5 mm/min), until 85% of the original height was reached (i.e., 21.25 mm). During the tests, the force–displacement relationship was recorded, from which the maximum compressive stress ($\sigma_{m}$), the stress at a 10% relative deformation ($\sigma_{10}$), and the compressive modulus (*E*) in the material’s proportional limit were determined. The obtained data were presented in the form of force-displacement graphs, allowing for the analysis of the material’s characteristics under compressive load. The results enabled the assessment of the influence of the structure and material properties on its mechanical behavior and allowed for conclusions regarding potential engineering applications.

### 2.4. Methodology of Compressive Strength Test Numerical Simulation

The finite element method (FEM) is a widely used computational tool for analyzing the mechanical behavior of structures, including deformation patterns, stress distribution, and failure mechanisms. In this study, numerical simulations were conducted to evaluate the mechanical response of a 3D-printed lattice structure subjected to uniaxial compression. The computations were performed using the explicit dynamic solver ANSYS Explicit Dynamics within the Ansys Workbench 2024 R2 (Ansys Inc., Canonsburg, PA, USA). This solver is particularly well suited for capturing large deformations as well as modelling failure initiation and propagation.

To optimize computational efficiency, a plane strain formulation was employed, justified by the uniform material properties across the specimen’s width and the consistent printing orientation along its length. Additionally, the elongated geometry of the sample supports the assumption that out-of-plane effects are negligible, allowing the analysis to focus on dominant in-plane deformations.

The investigated lattice structure consists of diagonally arranged unit cells, whose configuration significantly influences the mechanical response under compressive and impact loading. To accurately capture stress concentrations, deformation localization, and crack initiation, a structured quadrilateral mesh was utilized. The model was discretized using first-order quadrilateral elements with a size of 0.15 mm, determined through a mesh sensitivity study. The total number of elements in the analysis was 38,126 with 44,868 nodes for samples without resin and 76,365 elements and 77,449 nodes for simulations samples with resin.

**Figure 7:**
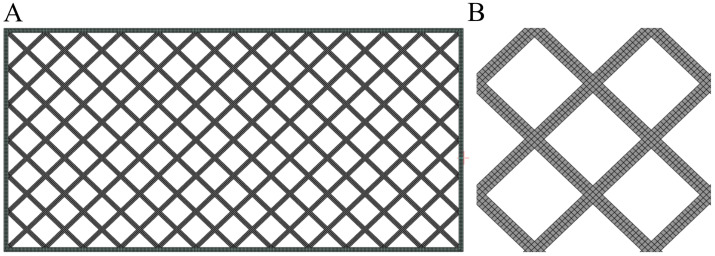
Numerical model: (**A**) entire model; (**B**) unit cell.

During the geometry preparation process, the size of the unit cells was adjusted to ensure proper alignment at the cell junctions, considering the absence of fillets and excess material necessary to maintain print continuity. The omission of these geometric details is a reasonable simplification in the context of numerical modelling, as it allows for efficient structural representation while preserving the key mechanical characteristics of the system. The discretized sample is shown below (Figure 7).

**Figure 8:**
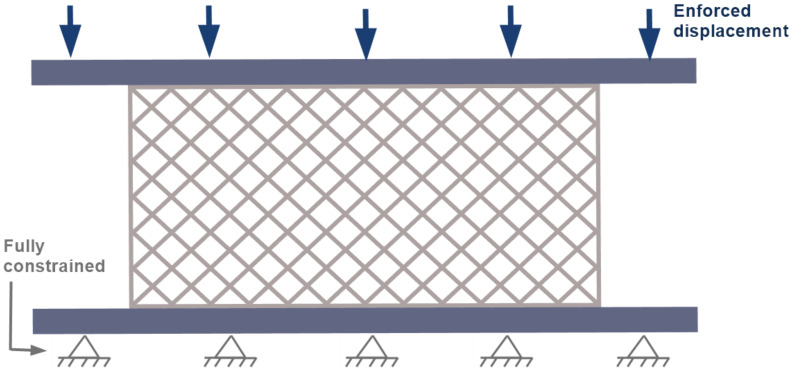
Initial boundary conditions of the numerical model during the compression test in the computational environment.

The specimen was positioned between two rigid steel plates. The bottom plate was fully constrained, restricting all translational and rotational degrees of freedom. The top plate was subjected to a displacement-controlled downward motion (Figure 8).

In this study, the material’s plastic response was characterized using a modified Johnson–Cook (J-C) constitutive model. The J-C model is widely employed in numerical analyses, primarily because its parameters can be determined with relative ease and it integrates smoothly with numerous finite element software packages (e.g., ABAQUS, LS-Dyna). Its broad adoption also stems from its ability to capture strain hardening and provide reliable predictions under a variety of loading conditions [30]. Recent research confirms the model’s effectiveness in numerical simulations, highlighting its high accuracy when reproducing the mechanical responses of diverse materials and processing conditions [31]. Furthermore, various optimization strategies have been developed to refine the J-C parameter set, enabling a closer match to experimental data and improving the model’s predictive capability [32].

Although the complete formulation of the model can incorporate strain rate and temperature dependencies, the present analysis focuses on quasi-static loading. Consequently, only the strain-hardening component is retained. This approach offers a consistent yet concise representation of the plastic behavior under the studied conditions, aligning with contemporary practices in numerical modelling and experimental validation of the J-C model [33].

(1) $$\sigma_{y}=\left(A+B{\bar{\varepsilon }}^{p^{n}}\right)\left(1+C\ln{\dot{\varepsilon }}^{*}\right)\left(1-T^{*m}\right)$$

where *A* is the initial yield strength of the material, *B* is the strain hardening coefficient, *n* is the strain hardening exponent, *C* is the strain rate effect, ${\bar{\varepsilon }}^{p^{n}}$ is the the equivalent plastic strain, ${\dot{\varepsilon }}^{*}$ is the effective strain rate (dimensionless), $T^{*}$ is the homologated temperature (dimensionless), and *m* is the thermal softening coefficient.

The Johnson–Cook parameters (A, B, and n) employed in this research were determined in the authors’ previous study [24], where static tensile tests were conducted on FDM-printed specimens. These parameter values (Table 2) were subsequently validated by comparing the simulated stress–strain curves with newly acquired experimental measurements, indicating that they satisfactorily capture the mechanical properties of the investigated structures.

## 3. Results

### 3.1. Results of Experimental Research

**Figure 9:**
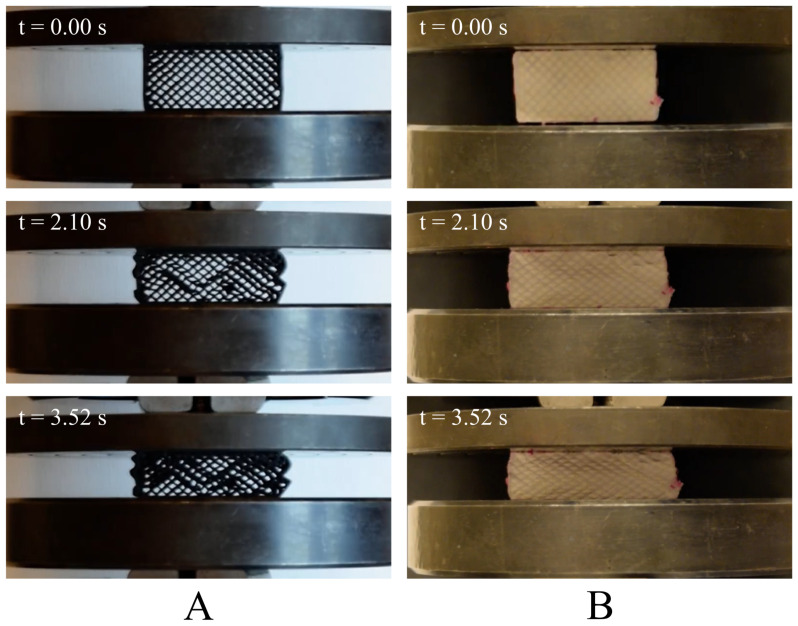
Results of the static compression test for different times (0.00 s; 2.10 s; 3.52 s): (**A**) PETG carbon sample with 30% infill; (**B**) PETG carbon sample filled with resin.

The mechanical properties of polymer materials under compression were analyzed based on the requirements of ISO 844, which describes methods for determining mechanical parameters under axial loading. The basis was to obtain force–displacement (F=f(x)) characteristics, from which the compressive modulus, compressive strength at 10% deformation, relative elongation, and compressive strength were determined. Below is the progressive compression process of the samples (Figure 9).

**Figure 10:**
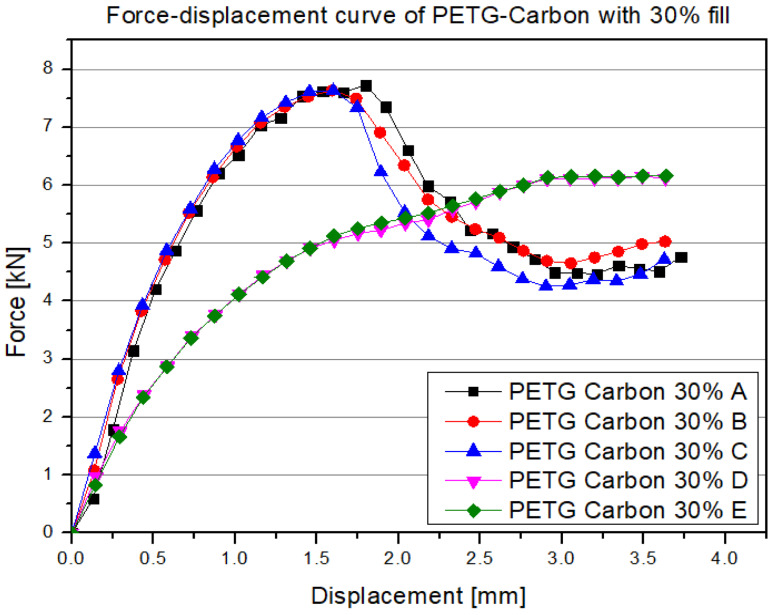
Results of the static compression test for selected structures made from PETG carbon with 30% infill.

The first material analyzed was PETG carbon with 30% infill (Figure 10).

In the initial phase of loading (approximately 0–1 mm of displacement), a rapid, almost linear increase in force is observed, indicating the dominance of elastic deformations. Despite having a partially hollow, “cellular” structure, the printed material is still intact in this range, and the print elements primarily behave elastically. The maximum force, usually occurring in the displacement range between 1.5 and 2 mm, represents the load-bearing capacity limit of the internal walls and the connections between filament layers.

**Figure 11:**
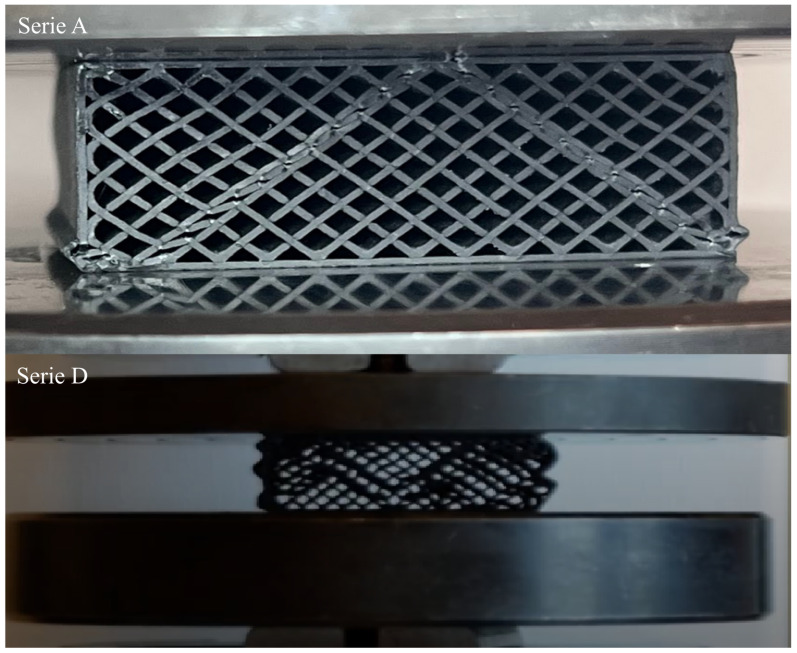
Difference in the deformation pattern of samples from series A (**top**) and D (**bottom**) made from PETG carbon.

For the discussed PETG carbon 30% samples, the peak values typically oscillate around 7–8 kN, with differences in the characteristics between series A, B and C and series D and E. These differences result from varying sample deformations during the test (Figure 11). The deformation differences stem from the specifics of the 3D printing process, where the nozzle diameter influences print accuracy. Print accuracy, in turn, affects the accuracy of infill lines and their connections to the sample’s contours. Distortion of the infill lines at the contour significantly impacts their interaction. After exceeding the maximum load point, the graph shows a characteristic quite sharp drop in force, indicating the onset of local damage and plastic deformation in the material’s structure.

**Figure 12:**
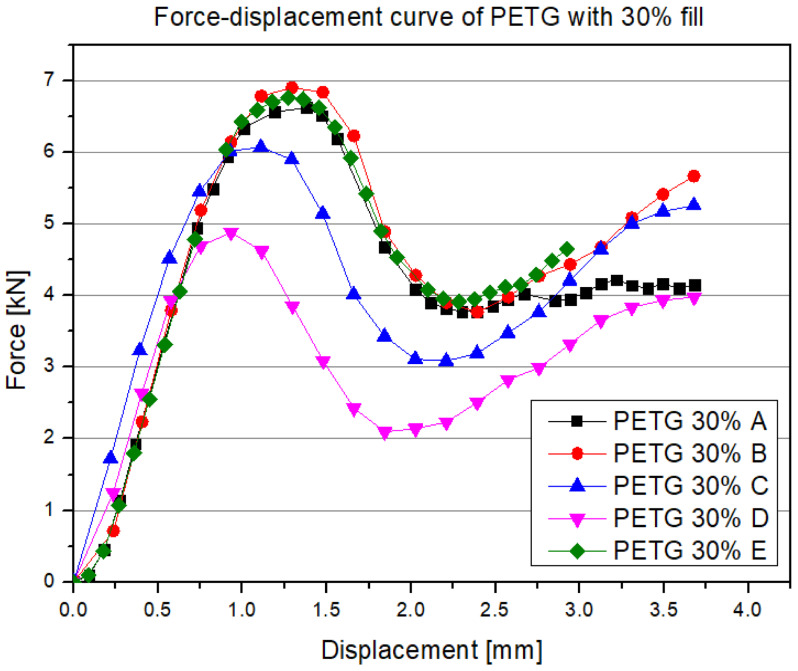
Results of the static compression test for selected structures made from PETG with 30% infill.

The top image (Figure 11, top view) shows a PETG carbon sample during the compression test. The bands of the highest stresses are visible, spreading out at an angle of approximately 40° relative to the base of the sample. These lines represent areas of stress concentration and local weakening of the structure, along which the destruction process begins. The lower photograph captures a different behavior under similar compressive loading, indicating a different form of deformation. This may result from varying print accuracies, which affect the way loads are transferred. It is worth noting that local weakening in specific areas of the structure can significantly influence the damage propagation path and thus shape the final course of deformation. These differences emphasize the importance of material, structural, and technological parameters in shaping compressive strength. The next material analyzed was PETG with 30% infill (Figure 12).

In the initial phase of loading (around 0–1 mm displacement), there is a rapid increase in force, which follows an almost linear pattern, indicating the dominance of elastic deformations. In this zone, all internal walls and the connections between filament layers work in the elastic range, without showing any local damage. After exceeding about 1 mm of displacement, a clear (though varied depending on the series) maximum force point appears, usually in the range of 5–7 kN. In general, the PETG 30% samples reach slightly lower peak values than the analogous carbon fiber-reinforced material (PETG carbon), yet still demonstrate relatively high load-bearing capacity. This maximum corresponds to the development of initial cracks or buckling of the walls, leading to the initiation of damage in the fill structure. After reaching the peak load, a sharp drop in force is observed, which is different for each sample, but generally exceeds 1–2 kN.

**Figure 13:**
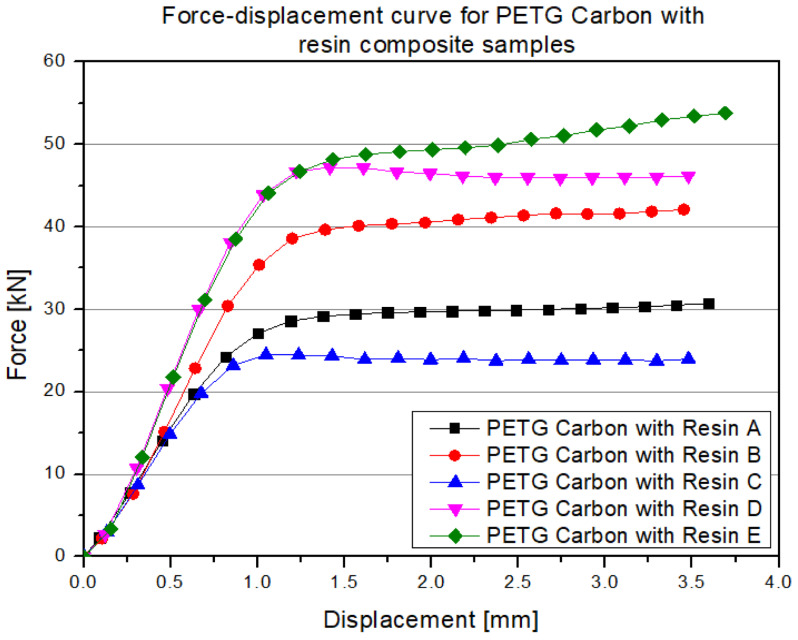
Results of the static compression test for selected structures made from PETG carbon filled with resin.

Compared to the PETG carbon series, all PETG samples exhibit similar work characteristics, though with some deviations. This may result from the nature of the material itself, which lacks carbon fiber inclusions that could disrupt both filament flow and nozzle coating due to friction. Below are the results for the samples made from PETG carbon with 30% infill and resin addition (Figure 13).

**Figure 14:**
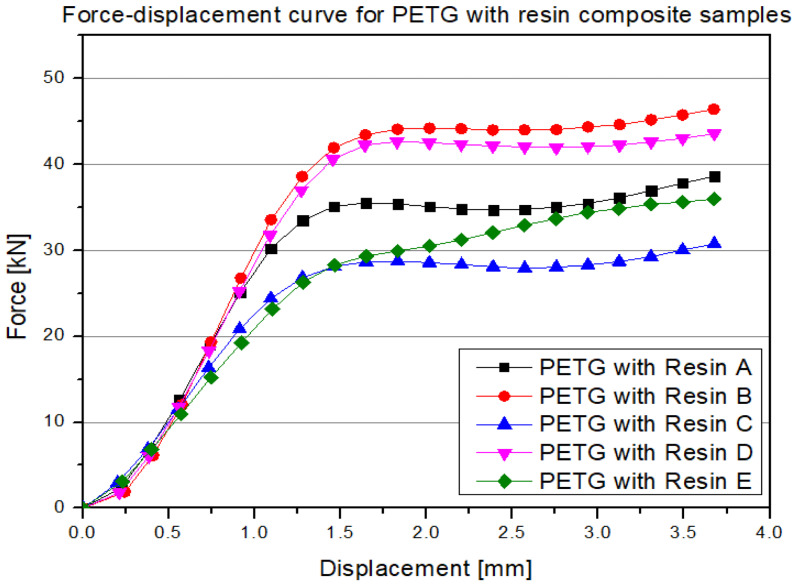
Results of the static compression test for selected structures made from PETG material filled with resin.

In the obtained force–displacement graph, in the first phase (usually up to around 1 mm displacement), a characteristic quasi-linear increase in force is observed, resulting from the elastic deformation of both the PETG carbon filament itself and the bonding of the resin with the internal walls. Then, in the 1–2 mm displacement range, a maximum load point is observed, whose value is significantly higher than in the case of the analogous unimpregnated sample. This occurs because the polyurethane resin structurally reinforces the walls and fill, reducing phenomena such as buckling or layer cracking in the printed structure. After reaching the peak force value, a relatively mild decrease in load can be observed, suggesting that even after local damage (e.g., cracking of individual layers), the resin acts as a "bridge" between the filament fragments, slowing down the process of complete structural collapse. As a result, a more extended plateau phase is achieved—the force remains at a relatively high level despite further displacement increases. This is a characteristic indicating higher resistance to crack propagation in materials with impregnated filling, compared to classic 3D printing. The final part of the graph may show a slight increase in force (or maintaining force at a higher level) when locally deformed areas of resin and filament begin to press against each other, further “blocking” further deformation. The differences in the shape and values of the curves between the individual samples result from the inhomogeneity of the resin infiltration process (e.g., trapping air bubbles, differences in curing time) and the typical 3D printing variations in adhesion or filament path geometry. Below are the results for samples made from PETG material with 30% fill and resin addition (Figure 14).

**Figure 15:**
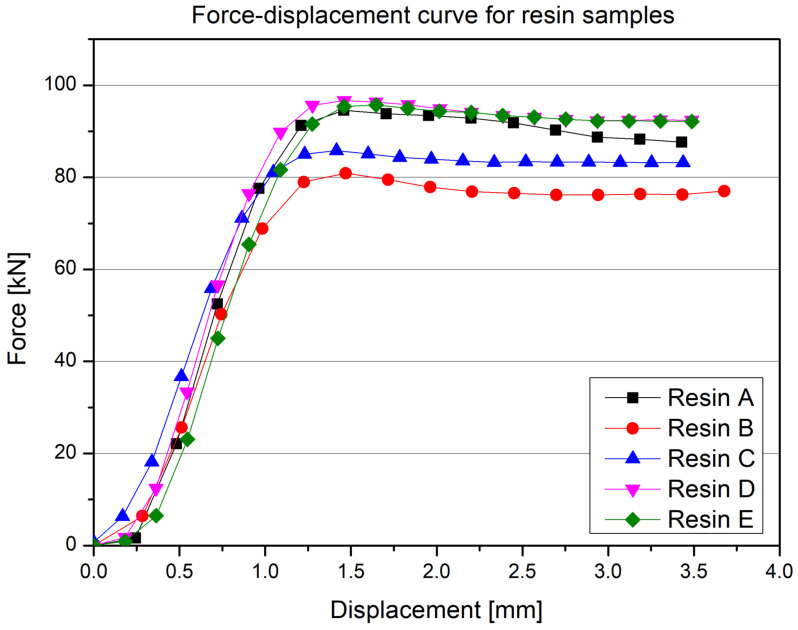
Results of the static compression test for the resin.

In the initial displacement range (around 0–1 mm), the force increases exceptionally steeply, suggesting a significant stiffening of the structure due to the filling of free spaces with resin. In the next stage (1–2 mm displacement), the curves in most samples reach values in the range of 30–50 kN, indicating the high load-bearing capacity of the composite formed by combining PETG and polyurethane resin. In contrast to classic cellular materials (e.g., printed solely with filaments), no sudden collapse is observed in these samples after exceeding the maximum force. Instead, the curves transition into a phase of relatively mild stabilization, with a slight but sustained increase in force values even as displacement continues to rise (up to 3–4 mm). The visible differences between the series (A–E)—including the values of the maximum forces (from about 30 kN up to 50 kN) and the slope of the curves at higher displacements—may result from the inhomogeneity of the impregnation process (differentiated resin penetration into the sample) as well as slight changes in 3D printing quality (such as layer adhesion or precise filament path geometry). However, all the curves generally confirm a significant improvement in mechanical properties compared to non-reinforced prints, while also showing a typical feature of composites: the absence of a sudden drop in load-bearing capacity after exceeding the peak value. The last material analyzed is the resin filling itself, the results of which are presented below (Figure 15).

**Figure 16:**
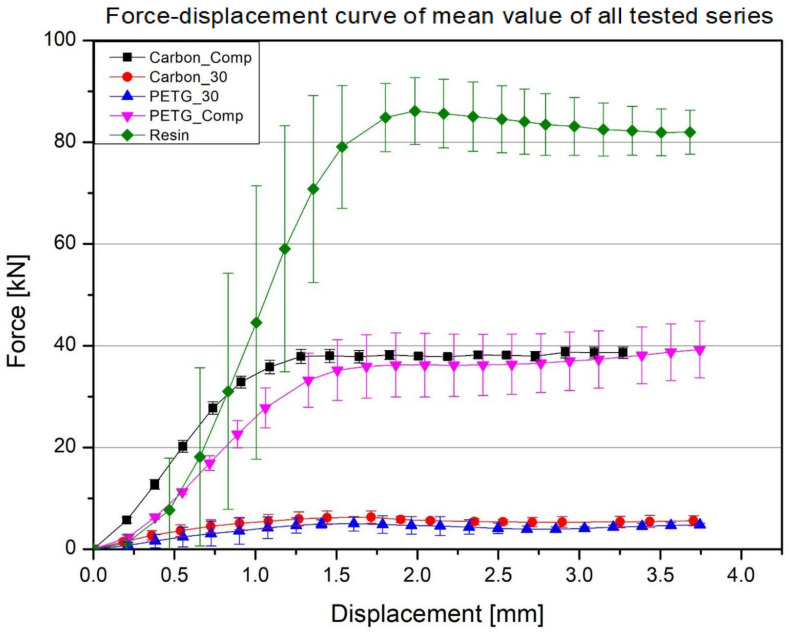
Averaged results of the static compression test for all series of samples.

The curves for each series (A, B, C, D, and E) indicate that at small displacements, the force increases very steeply, which is typical for rigid, fully solid polymer materials where microporosity or other internal defects do not play a significant role in the initial deformation phase. Above a displacement of around 0.5–1 mm, the curves transition into a plateau phase or a mild further increase, reaching maximum values of 80–100 kN. This relatively flat portion of the curve suggests that the material is still capable of bearing the load without abrupt failure mechanisms; instead, a gradual, small-scale internal deformation is observed. Differences between the individual samples (A–E) may stem from slight variations in the resin manufacturing process (e.g., ingredient ratio, curing conditions, homogenization), as well as from specific testing conditions (sample orientation, dimensional deviations). Nevertheless, the characteristic lack of sudden failure across the entire range of tested displacements distinguishes polyurethane resin from composites or 3D prints with infill—in comparison, those materials often show rapid drops in force after reaching the maximum. Below (Figure 16), the averaged results for all tested samples are presented.

At the bottom of the graph, there are curves for 3D-printed materials without impregnation, namely PETG 30% fill and PETG carbon 30% fill. These materials, due to their partially cellular structure and limited filling, reach relatively low force values (usually not exceeding several kilonewtons), after which, around 1–2 mm displacement, they transition into a reduced plateau phase. The addition of carbon fiber (PETG carbon) provides slightly higher loading values compared to pure PETG; however, these are still significantly lower than in the samples impregnated with resin. Much higher force values—in the order of several tens of kN—are achieved by the samples in which the structure of the printed material (PETG or PETG carbon) has been filled with polyurethane resin. The impregnation of the empty spaces and the bonding of the filament walls with the cured resin causes the composite to acquire the characteristics of a solid material, leading to a clear increase in both stiffness and load-bearing capacity in the compression test. The differences between the PETG with resin and PETG carbon with resin series mainly result from the reinforcement with carbon fiber and potential variations in the level and uniformity of resin saturation. Nevertheless, both versions of the composites significantly outperform the non-impregnated samples. The highest load values (above 80 kN) are achieved by pure polyurethane resin (Resin), whose solid, uniform structure does not contain typical 3D print pores or layers. This material reacts very rigidly in the initial loading phase, resulting in a nearly vertical increase in the force curve, and then maintains a high, relatively stable level, not showing the typical "break" characteristic of cellular materials. The material property parameters for each material and structure are summarized in the table below (Table 3).

### 3.2. Results of Numerical Simulations

**Figure 17:**
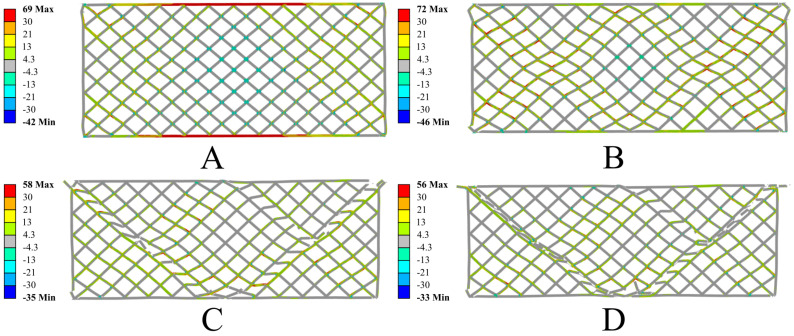
Results of the numerical simulations for PETG carbon. Maximum principal stress [MPa] in successive deformation steps. True scale deformation: (**A**) 1 mm jaw displacement; (**B**) 2 mm jaw displacement; (**C**) 3 mm jaw displacement; (**D**) 4 mm jaw displacement.

**Figure 18:**
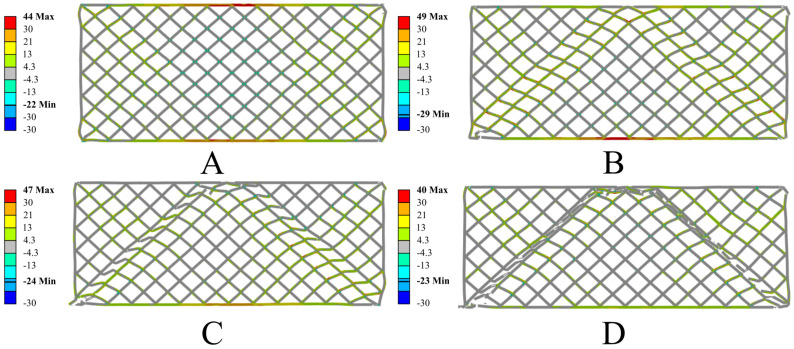
Results of the numerical simulations for PETG. Maximum principal stress [MPa] in successive deformation steps. True scale deformation: (**A**) 1 mm jaw displacement; (**B**) 2 mm jaw displacement; (**C**) 3 mm jaw displacement; (**D**) 4 mm jaw displacement.

The conducted numerical simulations showed high consistency with the experimental test results, effectively reproducing the deformation and failure mechanisms of the tested samples. The analysis of the results revealed a characteristic stress distribution leading to the initiation of damage. The degradation process of the structure began at the edges of the sample, where a triangular stress concentration zone formed, which then propagated toward the center of the sample. Additionally, in the initial loading phase, local buckling of the outer walls of the sample was observed at its base. This phenomenon resulted from the uneven distribution of compressive stresses and the interaction between neighboring lattice elements, indicating the presence of local geometric instabilities in the structure. In the case of the structure without resin impregnation, the largest stress concentrations occurred in the areas of connections between adjacent elementary cell segments. Elevated stress values in these regions led to crack initiation, which then propagated along these connections. As a result, the dominant failure mechanism was the local loss of material continuity along shear bands, which ultimately caused a gradual degradation of the entire structure’s load-bearing capacity. Below are the results of the numerical simulations for the PETG carbon structure (Figure 17) and the PETG structure (Figure 18).

**Figure 19:**
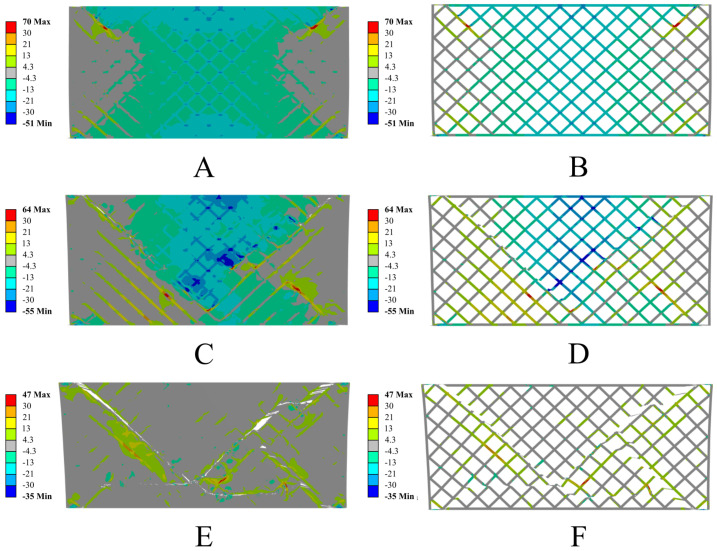
Results of the numerical simulations for PETG carbon with resin. Maximum principal stress [MPa] in successive deformation steps. True scale deformation: (**A**,**B**) 0.5 mm jaw displacement; (**C**,**D**) 0.75 mm jaw displacement; (**E**,**F**) 1 mm jaw displacement. On the left, the sample with resin filling is shown; on the right, only the printed structure is shown, without displaying the resin.

**Figure 20:**
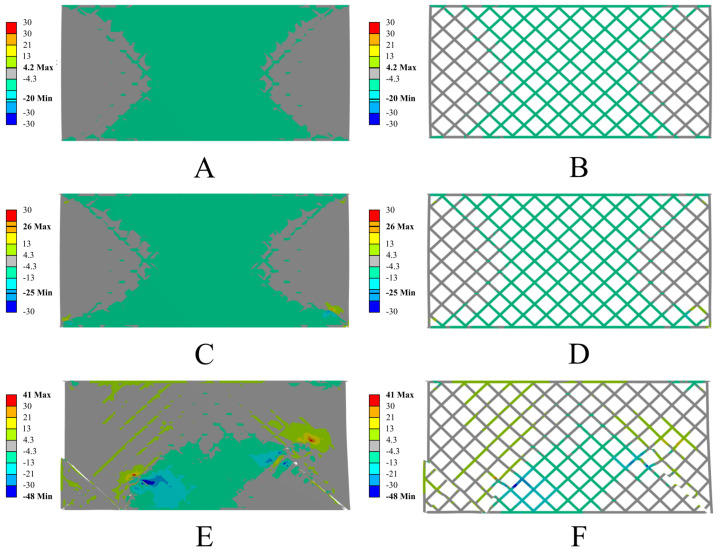
Results of the numerical simulations for PETG with resin. Maximum principal stress [MPa] in successive deformation steps. True scale deformation: (**A**,**B**) 0.5 mm jaw displacement; (**C**,**D**) 0.75 mm jaw displacement; (**E**,**F**) 1 mm jaw displacement. On the left, the sample with resin filling is shown; on the right, only the printed structure is shown, without displaying the resin.

The numerical analysis also revealed significant differences in the behavior of both structures, resulting from the different mechanical properties of the materials used. The structure made from PETG carbon exhibits higher stiffness and limited deformation in the initial loading phase, while the sample made from PETG shows greater susceptibility to deformation and does not fracture in a brittle manner, as is the case with the carbon-fiber-modified material. In the later stages of loading, a more noticeable deformation of the individual elementary cells in the PETG structure is observed compared to the sample with the addition of carbon, indicating significant differences in the deformation and destruction mechanisms between these materials. Below are the results of the numerical simulations for the PETG carbon structure with resin (Figure 19) and PETG (Figure 20).

The numerical simulations conducted are consistent with the experimental observations, where a clear end of the elastic range was observed at a jaw displacement of around 1 mm, after which the material begins to undergo plastic flow. In the numerical model, this is reflected by a sharp increase in local plastic deformations in the most stressed segments of the structure, which over time lead to crack initiation. As the load increases, other areas of the structure take on some of the forces until continuity is lost in the regions with the highest stress concentration. Comparative analysis indicates that samples made from PETG carbon exhibit higher stiffness in the initial phase of deformation, which results in a quicker increase in force at small displacements. At the same time, these samples have a lower allowable maximum plastic deformation, leading to the earlier initiation and propagation of cracks, and consequently, faster failure of the structure’s continuity. In the case of pure PETG, the plastic deformation spreads over a larger area, allowing for a longer period of load transfer before complete failure. Therefore, the comparison of experimental results with the modelling confirms that the addition of carbon fibers to PETG increases the material’s initial stiffness but simultaneously reduces the level of plastic deformation that the structure can sustain before losing its integrity.

### 3.3. Specific Energy Absorption

As part of the conducted analysis, calculations were performed to demonstrate how the specimens absorb the energy applied during the compression test. The widely adopted specific energy absorption (*SEA*) metric was employed in this context.

(2) $$SEA=\frac{EA_{abs}}{m}=\int_{0}^{\delta }\frac{Fd\delta }{m}$$

where $EA_{abs}$ represents the total energy absorbed by the specimen (e.g., under compression to a specified strain or failure), *m* denotes the mass of the specimen, *F* is the compression force, and *δ* is the compression distance. The SEA parameter thus indicates how much energy the material can absorb per unit mass. In the case of PETG and PETG carbon specimens printed at 30% infill, the initial mass is lower compared to a fully 3D-printed or a solid resin cast sample. However, the corresponding absorbed energy remains relatively low, owing to the lattice nature of the structure and weak inter-layer bonding.

Impregnating these specimens with resin substantially increases their mass, yet it also markedly enhances the total absorbed energy. This effect arises from filling the internal voids and creating a PETG–resin or PETG carbon–resin composite, which exhibits reduced susceptibility to localized crack initiation as well as higher strength and stiffness.

**Figure 21:**
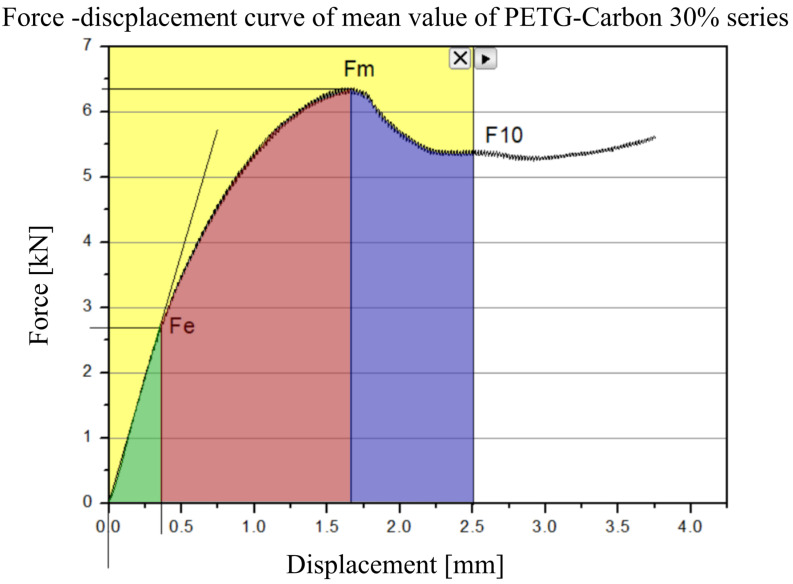
The measurement of the SEA parameter for the analyzed specimens was performed by integrating the force–displacement curve within three distinct intervals of deformation. Specifically, the green range corresponds to the purely elastic regime, the red range extends up to the yield point, and the blue range encompasses deformation up to 10% strain.

The SEA parameter was determined for all specimens across three distinct ranges (Figure 21). The green range corresponds to the elastic deformation regime, the red range extends up to the yield point, and the blue range captures deformation up to 10% strain. The resulting SEA values were calculated as averages for each of the tested sample types: PETG, PETG carbon, PETG resin, PETG carbon resin, and resin.

**Figure 22:**
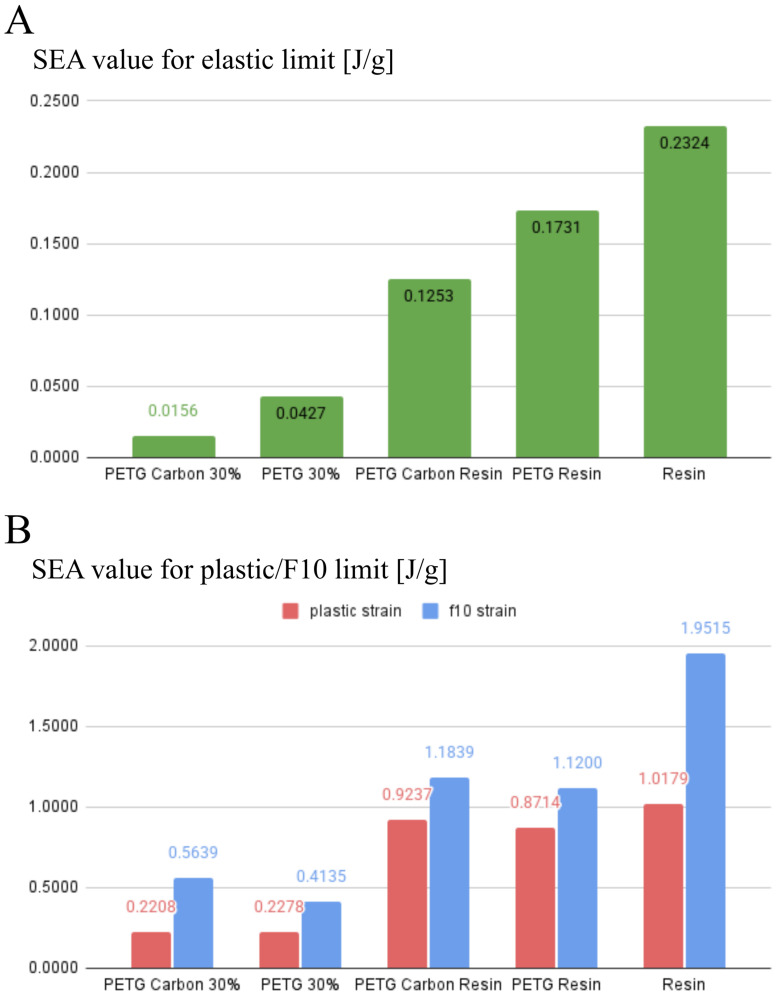
The energy absorbed by the specimens within the analyzed deformation intervals.

Figure 22 illustrates the energy absorbed by the specimens in the specified intervals. Chart A presents the energy per unit mass within the elastic range, whereas Chart B shows the corresponding values in the plastic range (red) and for deformation up to 10% strain (blue).

## 4. Discussion

The limited load-bearing capacity and stiffness observed in the case of conventionally printed PETG and PETG carbon samples (with 30% fill) are largely due to the porosity and anisotropic nature of 3D printing. This structure features numerous potential delamination planes and voids between filament paths, which results in relatively low maximum stress values (*σ*_*m*_ ≈ 2.5−3 MPa). Impregnation with polyurethane resin significantly improves these parameters by bonding the layers and filling the voids, resulting in a composite with greater homogeneity and higher strength. The partial "bridging" of cracks by the resin also leads to a less abrupt failure mechanism in the later stages of compression. PETG carbon, containing reinforcing carbon fibers, exhibited marginally better rigidity and resistance to initial deformation than standard PETG. The fibers helped to constrain deformation and provided localized reinforcement, but the overall compressive strength of both materials remained low due to the discontinuous nature of the filament paths and the porosity of the structure. Carbon fiber prints have somewhat better mechanical properties than pure PETG, but the decisive strengthening factor is the introduction of the resin. The resin’s low viscosity allowed it to flow into the internal lattice, filling gaps, bonding layers, and eliminating delamination planes. This created a composite that behaved more like a solid material rather than a layered or cellular structure. As a result, both PETG and PETG carbon samples became much more resistant to compressive loads. Not only did the resin significantly increase their ability to withstand higher forces, but it also altered the failure behavior. Instead of sudden collapse after peak loading—as observed in the unfilled samples—the resin-infused composites showed gradual, progressive deformation. The differences in maximum values between the PETG carbon with resin and PETG with resin can be explained by additional stiffening from the fibers, although this effect remains smaller than might be expected given the remaining porosity. On the other hand, the testing of samples made from 100% polyurethane resin confirms that, in the absence of layers and voids, this material achieves much higher maximum stress values (*σ*_*m*_ ≈ 36 MPa) and elasticity modulus (*E* ≈ 1.1 GPa). The infusion of polyurethane resin F8 had an interesting impact on the specific energy absorption (SEA) of both infused and non infused structures. In the elastic deformation range, the SEA of PETG samples increased approximately threefold after resin infusion, while that of PETG carbon saw an increase of about 2.5 times. This trend continued in the plastic region up to the yield point, where both materials demonstrated more than a threefold improvement in their energy absorption capacity per unit mass. Even when analyzed up to 10% strain, resin-filled samples absorbed significantly more energy than their non-infused counterparts. The SEA of PETG increased by nearly 2.7 times, and that of PETG carbon increased by around 2.5 times. Despite the added mass from the resin, the gain in absorbed energy was disproportionately higher, resulting in much better energy absorption efficiency. From a materials engineering perspective, the results indicate that while 3D printing allows for precise geometry shaping, the layers and internal void areas in the fill present a significant barrier to achieving high compressive strength in the sample. The numerical simulations allowed for the analysis of the global deformation pattern and destruction mechanism occurring in different areas of the tested structure. The numerical model incorporated geometric simplifications, including slight modifications of dimensions and the assumption of uniform cross-sections in each section of the sample. The omission of local constrictions and rounded edges resulting from the limited precision of additive manufacturing technology could significantly influence the actual degradation mechanism, leading to modifications in deformation paths and the initiation of alternative plasticization areas. Despite these simplifications, the numerical analysis successfully replicated the key aspects of the behavior of the tested structures and identified areas at higher risk for damage initiation. The regions most prone to degradation include the side walls of the sample and areas where the characteristic triangular stress concentration zones form. High values of principal stresses were recorded at the nodes along these areas, leading to gradual plasticization of the material and subsequent initiation of local cracks. As the degradation of further nodes in the structure progresses, the global deformation mechanism changes, determining the destruction propagation paths and the final load-bearing capacity of the system. Additionally, the numerical analysis revealed significant changes in the sample’s volume in the direction perpendicular to the compression axis. This phenomenon manifests as the development of substantial tensile stresses in the central frame area of the structure. The observed elongation of the sample in the transverse direction relative to the applied load leads to local stress concentration, which favors crack initiation in these areas. This process is particularly noticeable in regions symmetrically positioned on the top and bottom edges of the sample, where the first signs of material degradation appear. The development of these damages influences the further evolution of the destruction mechanism, contributing to the gradual loss of the structure’s load-bearing capacity. Similar observations were conducted in [34], which examines the energy absorption capacity and deformation mechanisms of 20 cellular topologies classified into four groups, employing numerical compression tests combined with experimental investigations on ABSplus specimens.

Although the base material used in this study differs significantly from stainless steel, ref. [35] employed a strut-based lattice with a comparable geometric layout and observed similar compression-related deformation modes. This finding implies that cell shape and topology—rather than material choice alone—principally dictate the early-stage performance of such structures. In particular, larger cell sizes promote more pronounced initial force peaks due to a stretch-driven load transfer mechanism (tensile and compressive forces within diagonal members); once this peak is exceeded, the structure undergoes progressive softening (post-yield), a phenomenon frequently noted in the literature [36]. Consequently, geometry-driven parameters—such as cell size and arrangement—exert a dominant influence on mechanical behavior, even under varying material conditions. When resin is partially or fully infused into the lattice, it occupies the internal cavities and stiffens regions that would otherwise be porous, altering the stress distribution and helping to reduce or eliminate the pronounced early force peak. This resin-based reinforcement bridges potential weak zones in the struts, diminishing the abrupt collapses characteristic of unfilled samples. The resulting composite exhibits significantly improved energy absorption capacity per unit mass compared with unfilled PETG or PETG carbon lattices. Nonetheless, because pure resin itself has the greatest mass, a substantial increase in total sample weight may be detrimental in certain applications. Furthermore, industrial-scale or semi-industrial filling processes can suffer from the incomplete infiltration of voids, which introduces structural inhomogeneity and reduced mechanical performance. Although using a vibration table improves resin penetration, it does not entirely resolve the problem of residual voids and uneven resin distribution. Consequently, balancing the mass increase against the mechanical benefits of resin infiltration remains a key consideration in designing such lattice–resin systems.

## 5. Conclusions

The conducted studies demonstrated a significant impact of polyurethane resin impregnation on the mechanical properties of samples made from PETG and PETG carbon materials using 3D printing. Both the analysis of force–displacement curves and the obtained values of the maximum stress (*σ*_*m*_) and elasticity modulus (*E*) unequivocally confirm a substantial increase in load-bearing capacity and stiffness with impregnation. Printed samples (both PETG and PETG carbon) filled with resin achieved maximum stress values (*σ*_*m*_) up to five times higher than the non-impregnated variants.

The force–displacement curves for the impregnated samples showed not only higher peak forces but also a much more stable post-peak behavior, with a gradual decrease or even plateau in force, indicating progressive failure rather than sudden collapse. This suggests that the resin not only reinforces the structure mechanically but also helps to bridge cracks and inhibit their propagation. The resin acted as a structural binder, eliminating internal voids and weak interlayer connections inherent in FDM prints. This resulted in a more cohesive and homogeneous material, capable of distributing loads more effectively and delaying the onset of failure.

The highest strength parameters were obtained for samples made entirely of polyurethane resin, which exhibited high stiffness (*E* ≈ 1.1 GPa). Thus, it can be concluded that in the context of designing compression-loaded elements, it is crucial to reduce the porosity and layering of the print or to compensate for it through impregnation, which significantly increases resistance to local damage. In the case of compressing non-impregnated samples, significant differences were observed in their behavior at the early stages of deformation. The structure made from PETG carbon exhibited higher initial stiffness, as confirmed by the steeper force increase in the first phase of loading. While traditional solid polymers (such as fully cast polyurethane or injection-molded plastics) typically exhibit high compressive strength and consistent, isotropic behavior, 3D-printed structures—especially with partial infill—are often limited by internal voids, weak layer bonding, and anisotropy. However, when resin is infused into printed PETG or PETG carbon lattices, the created composites offer a hybrid performance profile: they retain the geometric flexibility and lightweight nature of 3D-printed materials while gaining substantial strength, stiffness, and energy absorption characteristics more typical of solid polymers. The resin acts as a crack-bridging agent, slowing down the propagation of damage and extending the structure’s load-carrying capacity after initial failure points. These findings are supported by numerical simulations, which indicate that in the case of PETG carbon, there is no local buckling of the structural cells; instead, rapid failure occurs in regions of intense load flow.

The application of polyurethane resin to infiltrate additively manufactured lattice structures substantially augments their energy absorption capability. In practice, however, this approach can induce a notable increase in overall mass and result in uneven penetration of the internal pore network. By judiciously selecting resin viscosity, modifying cell geometry, and precisely regulating vibration parameters, one can optimize the infiltration process to balance added mass against gains in mechanical performance. Furthermore, to ensure the high reproducibility of samples and thorough resin saturation of the internal cavities, more advanced fabrication methods—such as vacuum-assisted processing—are recommended.

## Figures and Tables

**Table 1:** Material properties of plastics used in the study [23,24,25,26].

Material	Manufacturer	Density [g/cm^3^]	Hardness (Shore A/D)	Tensile Strength [MPa]	Elasticity Modulus [MPa]
PETG Carbon	Armor Group, Nantes, France	1.32	78.8	92.9	2071
PETG	TERVATIVE, Warsaw, Poland	1.26	70.0	84.6	970
PU Resin F8	Progmar, Leszno, Poland	1.25	75.0	25.0	20–300

**Table 2:** Material data used for numerical simulations [24].

Material	*E* [MPa]	*ν* [-]	*ρ* [kg/m^3^]	*A* [MPa]	*B* [MPa]	*n* [-]	Failure Strain [mm/mm]
PETG carbon	2071	0.4	1320	43.67	25.14	0.099	0.057
PETG	970	0.4	1260	33.57	17.40	0.262	0.105
Resin	677	0.4	1150	27.16	13.46	0.375	0.133

**Table 3:** Determined mechanical parameters as a result of static compression test of structures.

Material	*σ*_m_* [MPa]	*σ*_*e*_ [MPa]	*σ*_10_ [MPa]	*ε*_*m*_ [%]	*E* [MPa]
PETG carbon 30% fill	2.85 ± 0.29	1.03 ± 0.40	2.15 ± 0.15	8.79 ± 2.84	157.06 ± 12.74
PETG 30% fill	2.59 ± 0.38	1.76 ± 0.22	1.63 ± 0.40	5.94 ± 1.82	97.75 ± 9.55
PETG carbon with resin	16.10 ± 4.26	1.14 ± 0.28	1.53 ± 0.40	14.46 ± 0.47	60.97 ± 4.95
PETG with resin	15.71 ± 2.22	1.11 ± 0.19	1.45 ± 0.24	14.97 ± 0.02	44.63 ± 10.70
Resin	36.34 ± 2.53	26.98 ± 2.35	35.22 ± 2.78	6.53 ± 0.44	1117.22 ± 91.17

* *σ*_*m*_: maximum stress; *σ*_*e*_: stress at the elastic limit; *σ*_10_: stress at 10% strain; *ε*_*m*_: maximum strain; *E*: Young’s modulus.

## Data Availability

The original contributions presented in the study are included in the article, further inquiries can be directed to the corresponding author.
